# Systematic Review of Patient Outcomes and Associated Predictors After Microfracture in the Patellofemoral Joint

**DOI:** 10.5435/JAAOSGlobal-D-19-00151

**Published:** 2019-11-04

**Authors:** Jason B. Smoak, Melissa A. Kluczynski, Leslie J. Bisson, John M. Marzo

**Affiliations:** From the UBMD Department of Orthopaedics and Sports Medicine, Jacobs School of Medicine and Biomedical Sciences, State University of New York at Buffalo, Buffalo, NY.

## Abstract

We summarized the clinical outcomes and predictors of clinical outcomes after microfracture for chondral lesions in the patellofemoral joint (PFJ).

**Methods:**

Embase, PubMed, CENTRAL, BIOSIS, and CINAHL databases were searched between January 1, 1980, and January 1, 2019, to identify all articles that examined outcomes or predictors of outcomes of microfracture in patients with patellofemoral chondral lesions. Studies of full-thickness chondral lesions in the PFJ were included, whereas those involving adolescents, partial-thickness chondral lesions, and underlying patellar instability were excluded.

**Results:**

We found a total of 257 articles, of which 8 articles (174 patients) met our inclusion criteria. All studies found improvement in clinical outcomes after microfracture in the PFJ. Younger patients showed greater improvement in clinical outcomes than older patients. However, the effect of size, severity (grade), or location of chondral lesions on clinical outcomes after microfracture is unclear.

**Conclusion:**

We found improvement in clinical outcomes after microfracture in the PFJ at midterm follow-up. Age may be a predictor of successful outcomes and longevity of the repair; however, there is insufficient evidence regarding the influence of defect size, severity, and location on clinical outcomes.

Articular cartilage lesions in the knee are a common cause of knee pain, functional limitation, and disability.^1^ Approximately 60% of patients undergoing knee arthroscopy are found to have a chondral lesion(s) at the time of surgery, and about 36% of these lesions are found in the patellofemoral compartment.^2–4^

The management of articular cartilage defects in the patellofemoral joint (PFJ) remains a challenging task for orthopaedic surgeons. Typically, nonsurgical management, such as medications, injections, and physical therapy, is recommended for at least 3 months with the aim of restoring function and relieving pain.^5^ Surgical management may be considered if nonsurgical management fails. The combination of limited articular cartilage healing capacity and the complex biomechanics of the PFJ makes a formidable environment for chondral repair.^6^ The technique of microfracture popularized by Steadman has long been considered the benchmark to treat chondral defects in the knee.^7–9^ A systematic review by Shanmugaraj et al^10^ found microfracture to be the second most common cartilage restoration technique in the PFJ, second only to autologous chondrocyte implantation in the past 5 years (29.6% versus 45.5%). Other popular techniques include osteochondral autograft transfer and autologous matrix–induced chondrogenesis. Limitations of these techniques include donor-site morbidity, high cost, graft hypertrophy, and multiple surgeries.^11,12^ Microfracture has the advantage of being low cost, technically simple, and free of donor-site morbidity.^9,13^ Several studies have demonstrated pain relief and improved function and activity levels with microfracture in the knee.^1,13–15^

Although many studies have evaluated the outcomes and predictors of success for microfracture in the knee, very few studies have separated and reported results in the PFJ.^7,15,16,17,18,19,20,21,22^ Patellar cartilage is the thickest cartilage in the human body, with structural properties differing from the neighboring cartilage on the femoral condyles, and the PFJ also experiences different stresses compared with the tibiofemoral joint including high sheer forces.^15,23^ The outcome may differ for microfracture in the PFJ compared with the tibiofemoral joint and should be examined separately.

The aim of this systematic review was to summarize the literature that has examined the clinical outcomes and predictors of successful clinical outcomes of microfracture for articular cartilage defects in the PFJ. We hypothesized that patient outcomes would improve after microfracture of patellofemoral chondral lesions and that outcomes would be better in younger patients and in those with smaller patellofemoral chondral lesions.

## Methods

### Search Strategy

A systematic review of the literature was performed with Embase, PubMed, CENTRAL, BIOSIS, and CINAHL databases between January 1, 1980, and January 1, 2019, to identify all articles that examined outcomes or predictors of outcomes of microfracture in patients with patellofemoral chondral lesions. Search terms included microfracture OR marrow stimulation OR drilling in combination with patella OR patellar OR trochlea OR trochlear OR patellofemoral AND chondral OR cartilage. The reference lists of articles meeting inclusion criteria were manually reviewed to search for further applicable studies. The literature search was performed separately by two independent reviewers, and the results were compared. A flow diagram of the literature search was created in accordance with the Preferred Reporting Items for Systematic Reviews and Meta-Analyses statement.

### Selection of Studies

We included studies of patients with PFJ chondral lesions treated with microfracture, full-thickness cartilage defects, and adult patients with closed physes. Only studies that reported outcomes and/or predictors of outcomes of microfracture for PFJ chondral lesions were included. We excluded studies that did not involve microfracture or included patients with concomitant multiligamentous knee repair, patellofemoral malalignment, failed prior chondral repair, partial-thickness defects, knee instability, osteoarthritis, history of patellofemoral instability, and concomitant patellofemoral stabilization at the time of microfracture. We also excluded studies that did not stratify outcomes by the location of chondral lesion because the results pertaining specifically to patellofemoral chondral lesions could not be extracted, studies with incomplete data, or those not published in English.

### Data Extraction

The following data were extracted from each article: duration of postoperative follow-up, study design, level of evidence, location of chondral lesion (patella, trochlea, or patellofemoral), age, sex, body mass index (kg/m^2^), size of chondral lesion (cm^2^), histology results (fibrocartilage and hyaline cartilage), and postoperative outcome measures (International Cartilage Repair Society [ICRS] score, International Knee Documentation Committee [IKDC] score, global scoring systems, Lysholm score, and Tegner score). Two authors independently scored the methodological quality, including risk of bias, for each study with the Downs and Black Study Quality Assessment Tool.^24^ We modified this assessment tool by excluding questions pertaining to randomized controlled trials because our literature search did not produce any such studies (Table 1). The maximum scores, indicating good quality, were 9 for case series and 15 for observational studies. Data could not be pooled for meta-analysis because of heterogeneity in outcome measurement tools between studies.

**Table 1:** Downs and Black Criteria for Study Quality Assessment

Downs and Black Question	Balain et al^21^	Gobbi et al^20^	Kreuz et al^16^	Kreuz et al^19^	McCarroll et al^18^	Petri et al^15^	Steadman et al^9^	Zorman et al^17^
1	1	1	1	1	1	1	0	0
2	1	1	1	1	1	1	1	0
3	1	1	1	1	1	1	1	1
4	—	—	—	—	—	—	—	—
5	—	—	—	—	—	2	—	—
6	1	1	1	1	1	1	1	1
7	1	1	1	1	0	1	0	0
8	—	—	—	—	—	—	—	—
9	—	—	—	—	—	—	—	—
10	1	0	0	0	0	0	0	0
11	0	1	1	1	1	1	1	1
12	—	—	—	—	—	1	—	—
13	—	—	—	—	—	—	—	—
14	—	—	—	—	—	—	—	—
15	—	—	—	—	—	—	—	—
16	—	—	—	—	—	—	—	—
17	—	—	—	—	—	—	—	—
18	1	1	1	1	1	1	1	1
19	—	—	—	—	—	—	—	—
20	1	1	1	1	0	1	0	0
21	—	—	—	—	—	1	—	—
22	—	—	—	—	—	1	—	—
23	—	—	—	—	—	—	—	—
24	—	—	—	—	—	—	—	—
25	—	—	—	—	—	1	—	—
26	—	—	—	—	—	—	—	—
27	—	—	—	—	—	—	—	—
Total	8	8	8	8	6	14	5	4

## Results

**Figure 1:**
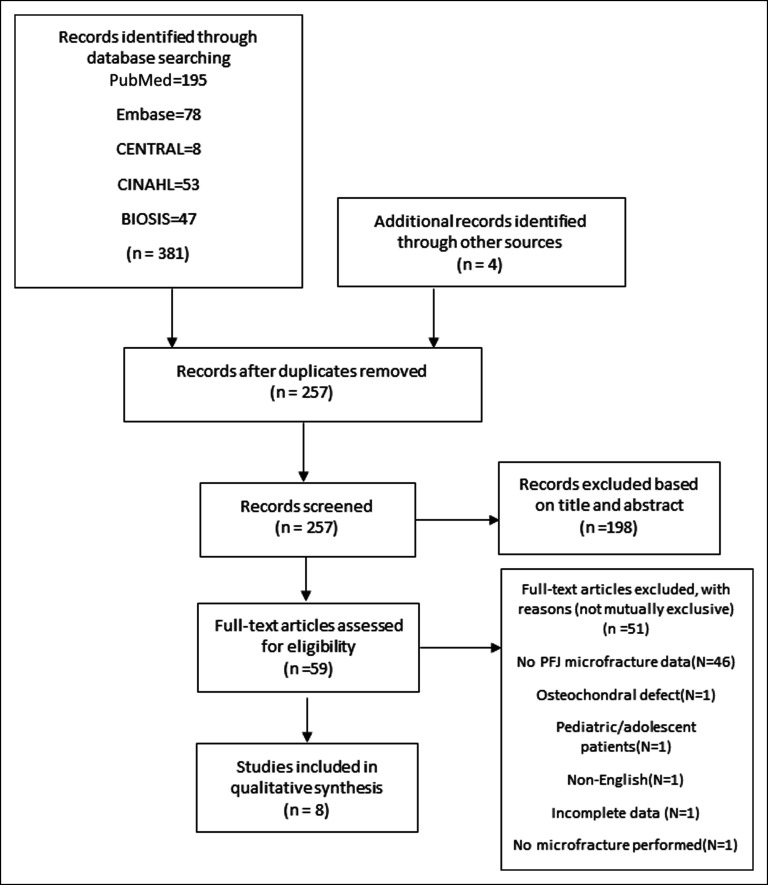
PRISMA flow chart. PFJ = patellofemoral joint

We found 257 unique articles after removal of duplicates, of which 198 were excluded based on title and abstract (Figure 1). Fifty-nine articles were reviewed in more detail, of which 51 were excluded and 8 studies were included in this systematic review. Two studies involved the same patient population, and both of these studies were included in our review.^16,19^ Of the articles reviewed in this study, there were two level II studies, two level III studies, and four level IV studies. Study quality according to Downs and Black criteria are presented in Table 1.^24^ Some Downs and Black criteria did not apply to most studies because they were not randomized controlled trials. Most studies had well-defined aims (Q1), outcomes (Q2), and patient characteristics (Q3). Most studies appropriately documented main findings (Q6), random variability (Q7), source population (Q11), and internal validity (Q18 and Q20), but probability values (Q10) and confounding (Q25) were not presented or addressed in most studies. Overall, study quality was good based on the total Downs and Black scores (maximum of 9 for case series and 15 for observational studies).

A total of 174 patients from all 8 studies had chondral lesions in the PFJ (Table 2). Chondral lesions were located in the patella in two studies, trochlea in two studies, trochlea and patella in two studies, and the PFJ (without specification for the patella or trochlea) in two studies. The average age of patients ranged from 21 to 49.4 years. The average size of cartilage defects ranged from 1.3 to 3.6 cm^2^, and the average postoperative follow-up ranged from 12 to 72 months.

**Table 2:** Study Characteristics and Results

Factors	Zorman et al^17^	McCarroll et al^18^	Steadman et al^9^	Gobbi et al^20^	Kreuz et al^16^	Kreuz et al^19^	Balain et al^21^	Petri et al^15^
Level of evidence	4	3	4	2	4	4	3	2
Sample size, N PFJ/N total	18/24	37/184	4/35	1/53	27/85	27/85^a^	50/193	10/17
Age (yrs)	Mean = 28.4 (range: 14-60)	Mean = 26 (range: 14-80)	Mean = 32.8 (range: 28-36)	Mean = 21	Mean_trochlea_ = 41.6 (range_trochlea_ = 26-55)	Mean_>40_ = 49.4 (range_>40_ = 42-55)	Mean = 40.6 (range: 16-76)	Mean = 41.7 (SD = 13.2)
					Mean_retropatellar_ = 38.5 (range_retropatellar_ = 23-55)	Mean_≤40_ = 34.1 (range_≤40_ = 23-40)		
Sex (male:female ratio)	13:11^d^	129:55^d^	4:0	1:0	Trochlea = 8:8	>40 yrs = 6:5	149:44^d^	6:4
					Retropatellar = 5:6	≤40 yrs = 7:9		
Chondral defects								
Location	Patella	Patella	Trochlea	Trochlea	N = 16 trochlea	Trochlea and patella	Patellofemoral joint	Patellofemoral joint
					N = 11 retropatellar			
Size (mean, range)	1.3 cm^2^ (0.5-2.5)^d^	NR	3.56 cm^2^ (1.5-6)	3.5 cm^2^ (NR)	Trochlea: 2.31 cm^2^ (1-4)	>40 = 2.39 cm^2^ (1.5-3)	NR	3.0 cm^2^ (NR)
					Retropatellar: 2.0 cm^2^ (1–3)	≤40 = 2.38 cm^2^ (1.5-4)		
Indications for surgery	Grade II (N = 6) or III (N = 18) chondral lesions based on Ogilvie-Harris classification	Grade II (N = 33)/III (N = 35) or IV (N = 2) chondral lesions based on Outerbridge classification	Full-thickness chondral lesions	Full-thickness chondral lesions	Grade III A/B chondral lesions based on ICRS	Grade III A/B chondral lesions based on ICRS	Full-thickness chondral lesions	Grade III/IV chondral lesions based on ICRS
Average follow-up, months (mean, range)	12 (5-17)^d^	46.8 (6-180)^d^	54 (24-168)^d^	72 (36-120)^d^	36 (NR)^d^	36 (NR)^d^	37 (NR)^d^	36 (NR)^d^
Outcome measures	Relief of preoperative pain (ie, able to resume motor activity identical to that of pretraumatic situation)	15-point satisfaction questionnaire (≥10 points is satisfactory)	Lysholm score	Tegner and IKDC scores	ICRS and Cincinnati scores	ICRS and Cincinnati scores	Lysholm, IKDC 1 (subjective), and IKDC 2 (symptoms)	Lysholm, Cincinnati, and IKDC subjective scores at 36 mo
		Or, combined interview and examination (≥16 points is satisfactory)				MRI defect filling	VAS global effect (preoperative and postoperative composite score of Lysholm, IKDC, and VAS using rank sum for paired data)	
						MRI score (cartilage signal, subchondral edema, and effusion)	Patient satisfaction (yes/no/cannot say)	
Results	Grade II (partial-thickness) lesions: 83% experienced relief of preoperative pain.	Patient satisfaction was reported in 76% (25/33) with grade II lesions, 64% (23/35) with grade III lesions, and 0% (0/2) with grade IV lesions. Satisfactory results were achieved in 67.6% of patients based on examination and interview and in 81.7% based on questionnaire alone for all patients in the microfracture group.^b^	Lysholm score improved from preoperative (mean = 56.8, range: 37–73) to postoperative (mean = 85.3, range: 80–95) assessment.	IKDC scores improved from C (abnormal) to B (nearly normal) in about 70% of patients. Tegner scores improved from 3 to 4 from preoperative to postoperative assessment.	Trochlear lesions: ICRS scores improved by 0.82 points (*P* = 0.006) and Cincinnati scores improved by 1.06 points (*P* = 0.01) from preoperative to postoperative assessment.	≤40 yrs old: ICRS and Cincinnati scores improved significantly more in patients aged 40 yrs or younger than those older than 40 yrs (*P* < 0.01).	Global effect size differed between PFJ (1.64) and medial (1.0) compartments (*P* < 0.05). Patient satisfaction was reported in 80% of those with PFJ lesions, 62% with medial lesions, and 82% with lateral lesions.	Lysholm postoperative scores at 36 mo were mean = 59.6 (SD 26.3).
	Grade III (full-thickness) lesions: 87% experienced relief of preoperative pain				Retropatellar lesions: ICRS scores improved by 0.73 points (*P* = 0.046) and Cincinnati scores improved by 1.45 points (*P* = 0.005) from preoperative to postoperative assessment.	Significant deterioration of ICRS and Cincinnati scores occurred between 18 and 36 months (*P* < 0.05).	^c^Lysholm sores improved from preoperative (mean = 55, range = 35–73) to postoperative (mean = 83, range = 67–93).	Cincinnati scores at 36 months Mean = 53.8 (SD 30.5).
					Femoral condyle lesions: ICRS scores improved by 1.4 points (*P* < 0.0001) and Cincinnati scores improved by 2.34 points (*P* < 0.0001) from preoperative to postoperative assessment.	>40 yrs old: ICRS and Cincinnati scores significantly improved over 36 mo (*P* < 0.05).	^c^IKDC 1 scores improved from preoperative (mean = 4, range = 3–5) to postoperative (mean = 2, range = 1–3).	IKDC subjective scores at 36 mo Mean = 50.1 (SD 24.9).
					Tibial lesions: ICRS scores improved by 0.91 points (*P* = 0.008) and Cincinnati scores improved by 1.45 points (*P* = 0.008) from preoperative to postoperative assessment.	Significant deterioration of ICRS (*P* < 0.05) and Cincinnati (*P* = 0.007) scores occurred between 18 and 36 mo, which was more pronounced than the deterioration in younger patients.	^c^IKDC 2 scores improved from preoperative (mean = 5, range = 1–9) to postoperative (mean = 0, range = 0–3).	There were no notable differences in any score between the MACT group and microfracture group at 36 mo.
					MRI defect filling score was significantly better for femoral condyle lesions compared with all other locations (*P* < 0.02).	MRI 36 months after surgery revealed better defect filling and better overall score in patients 40 years or younger compared with older patients (*P* < 0.05).	^c^VAS scores improved from preoperative (mean = 6.7, range = 4.6–7.9) to postoperative (mean = 1.4, range = 0.6–4.3).	

ICRS = International Cartilage Repair Society, IKDC = International Knee Documentation Committee, MACT = matrix-associated autologous chondrocyte implantation technique, NR = not reported, PFJ = patellofemoral joint, VAS = visual analog scale

a Same patient population.

b Results include partial-thickness lesions.

c Results include all compartments.

d Includes all patients.

### Clinical Outcomes

All the studies that were reviewed demonstrated improvement in all clinical outcome measures after microfracture in the PFJ (Table 2). Three studies showed improvement in Lysholm scores,^7,15,21^ three studies showed improvement in Cincinnati scores,^15,16,19^ and three studies demonstrated improvement in IKDC scores.^15,20,21^ The two studies by Kreuz et al showed clinical improvement in ICRS scores.^16,19^ Balain et al^21^ found improvement in the visual analog scale (VAS) for pain as well as a composite score of Lysholm, IKDC, and VAS for pain which they termed the global effect size. Two studies found that most patients were satisfied with their results after surgery.^18,21^ Zorman et al^17^ reported relief of preoperative pain and ability to resume motor activity identical to that of the pretraumatic situation in the majority of their patients. Gobbi et al^20^ found improvement in the Tegner score after microfracture in the PFJ. Finally, Kreuz et al calculated an MRI defect filling score 36 months postoperatively that was based on cartilage signal, subchondral edema, and effusion (Table 1).^16,19^

### Predictors of Clinical Outcomes

Kreuz et al^19^ found greater improvement in ICRS scores, MRI defect filling, and overall MRI score in patients aged 40 years or younger compared with patients older than 40 years undergoing microfracture for patellar or trochlear lesions (Table 1). ICRS and Cincinnati scores declined between 18 months and final follow-up at 36 months in patients of all ages, with the greatest decline occurring in patients older than 40 years.^19^ Balain et al^21^ also observed a trend of better outcomes in younger patients. They were unable to find a clear age cutoff, suggesting a more linear relationship between age and outcomes.

Two studies examined the effect of severity of chondral lesion on the outcome of microfracture. McCarroll et al^18^ found patient satisfaction after microfracture in 76% of 33 patients with Outerbridge grade II lesions, 64% of 35 patients with Outerbridge grade III lesions, and 0% of 2 patients with Outerbridge grade IV lesions. Zorman et al^17^ graded chondral lesions according to the Ogilvie-Harris classification and found that 83% of patients with grade II and 87% with grade III chondral lesions experienced relief from preoperative pain.

Two studies compared outcomes after microfracture by anatomic location of chondral lesions. Kreuz et al^16^ found that patients with chondral lesions on the femoral condyles had notably improved ICRS and Cincinnati scores compared with lesions on the tibial plateau, patella, or trochlea after undergoing microfracture. According to a composite score calculated from MRIs conducted at 36 months postoperatively, the best defect filling was seen in femoral condyle lesions (1.69 ± 0.74) treated with microfracture compared with tibial (2.64 ± 0.92) and patellofemoral defects (trochlear 2.56 ± 0.96 and patellar 2.55 ± 0.93). Balain et al found that patient satisfaction was observed for 80% of patients who underwent microfracture in the PFJ or lateral compartment and 62% who underwent microfracture in the medial compartment. Although satisfaction ratings were not statistically significant, when comparing global effect size (a composite score of IKDC, Lysholm, and VAS for pain), there was a significant improvement in the PFJ group compared with the medial compartment (*P* < 0.05).^21^

The average size lesion for all the studies included in this analysis was 2.56 cm^2^. None of the studies in this review examined the effect of lesion size on clinical outcomes.

## Discussion

Our review found evidence to suggest that patients have improved clinical outcomes after microfracture of symptomatic patellofemoral chondral lesions at midterm follow-up. Our review also found some evidence to suggest that younger patients may have improved clinical outcomes that are more durable over time compared with older patients. However, we could not draw any definitive conclusions regarding the effect of location, size, or severity of the chondral lesion.

Kreuz et al^19^ found that patients aged 40 years or older had improved outcomes compared with those younger than 40 years after microfracture in the PFJ, favoring the theory that younger patients have greater regenerative capacity. They also demonstrated worse outcome scores in all patients between 18 and 36 months postoperatively that was notably less pronounced in younger patients. Balain et al^21^ found an association of older age with lower satisfaction and poorer outcome as well. They found difficulty identifying an age cutoff and did not present any analysis; however, their study was underpowered to detect any differences based on age. Our review was unable to compare the outcomes of microfracture with age across the studies because of heterogeneity of outcome measures; however, the results published by Kreuz et al^19^ do suggest that functional outcomes may be superior and more durable in patients aged 40 years or younger compared with those older than 40 years.

Few studies have examined differences in the outcome of microfracture based on the location of lesion within the knee. Kreuz et al^16^ investigated differences between the femoral condyles, tibial plateau, patella, and trochlea. They found that femoral condyle lesions were associated with greater improvement in functional outcomes and MRI scores compared with the other locations. They also observed deterioration in the functional scores of patellar, trochlear, and tibial lesions between 18 and 36 months after surgery. This deterioration was not present in femoral condyle lesions. Balain et al,^21^ however, showed greater satisfaction in patients who underwent microfracture in the lateral compartment or the PFJ compared with the medial compartment. Outcome scores (Lysholm, VAS, and IKDC) were not reported for each compartment individually; however, a composite score generated from the outcomes showed a notably greater improvement of PFJ and lateral compartment lesions compared with medial compartment lesions. The use of a composite score limited our ability to compare the results to other studies. This study also did not report the size of the lesions which were treated, and Kreuz et al^16^ did not report the location of the femoral condyle lesions (medial or lateral). Differences in lesion size and location of femoral condyle lesions could in part explain the different results seen between tibiofemoral and patellofemoral lesions in both of these studies. Gobbi et al^20^ performed biopsies on second-look procedures and described a hybrid-like tissue with areas of both fibromyxoid tissue and initial hyaline transformation. The biopsies they performed were on the repair tissue in all compartments, and comparisons were not made between the samples. Studies have shown differences in stiffness of hyaline and fibrocartilage, which some authors suggest may lead to fissures in the surface texture and ultimately osteoarthritis.^25,26^ The different sheer and axial forces seen between the PFJ and tibiofemoral joint may play a role in the variability seen between compartments by virtue of the different loads placed on the repair tissue.

McCarroll et al^18^ were able to show improved outcomes of “trephine and drilling” with less severe chondral lesions. Although the authors did not refer to their technique as microfracture, the methods described a procedure that is now the commonly described technique for microfracture. Patients were grouped according to a modified Outerbridge classification, and upon review of their classification scheme, some, if not all, grade II lesions were likely partial thickness. Nonetheless, grade III and grade IV lesions were full thickness, and grade III lesions did have greater satisfaction compared with grade IV lesions. There were 33 and 35 patients in the grade II and III groups, respectively, yet only 2 patients with grade IV lesions who underwent microfracture as the authors opted for more extensive procedures in this group. Both grade IV patients had unsatisfactory results. The skewed distribution of patients among groups and likely inclusion of partial-thickness lesions make us question any correlations and conclusions drawn from this study pertaining to microfracture of the PFJ. Zorman et al^17^ also operated on grade II (partial-thickness) and III (full-thickness) lesions using Ogilvie-Harris classification, but they showed no notable differences in outcomes. The results of both studies were included in this review despite including partial-thickness defects for two reasons.^17,18^ First, because the results of full-thickness lesions were presented separate from the partial-thickness lesions and, second, to demonstrate any trends observed in outcomes. Both of these studies used different scoring measures, and thus, no comparison could be made between studies.

An articular cartilage lesion of up to 4 cm^2^ is an indication for microfracture in the knee, and many authors suggest a cutoff closer to 2 cm^2^ for the PFJ.^27,28^ Several authors recommend alternative procedures such as autologous chondrocyte implantation for lesions >2 to 3 cm in the PFJ because of concerns about durability and longevity of the repair tissue generated by microfracture in lesions of that size.^27–29^ Our study was unable to substantiate those recommendations for the PFJ. None of the studies that we reviewed were powered to detect differences in outcomes based on the size of the chondral lesion. In addition, the heterogeneity of outcome measures precluded an analysis between studies.

There are several limitations that we identified in our review. First, the heterogeneity of both subjective and objective outcome measures across studies precluded any meta-analysis. The lack of a standardized postoperative rehabilitation protocol across studies is a potential confounding variable, as several studies had different weight-bearing and range-of-motion protocols. The average follow-up period for the studies we reviewed was 41 months, and follow-up periods were highly variable, which also limited our ability to compare studies. This study did not find any long-term follow-up for microfracture in the PFJ, and thus, we were unable to draw any conclusions about the long-term effectiveness of microfracture, specifically in the PFJ. Finally, the quality of evidence we reviewed and the small number of overall patients are additional drawbacks.

## Summary

Our review found evidence to suggest that patients have improved clinical outcomes after microfracture in the PFJ at midterm follow-up. There is some evidence to suggest that age may be a predictor of successful outcome and longevity of the repair. We were unable to draw any conclusions regarding the influence of size, severity, and location of the chondral defect on the clinical outcome. This study provides the most comprehensive and current review of the literature pertaining to outcomes and predictors of outcomes after microfracture, specifically in the PFJ. Well-designed longitudinal studies with individual analysis of the PFJ are needed to deepen our understanding of cartilage restoration, specifically microfracture, in the PFJ.
